# Primary health care: Nurses’ meanings in a Colombian municipality

**DOI:** 10.15649/cuidarte.2487

**Published:** 2023-12-19

**Authors:** Lina Karina Bernal-Ordoñez, Juliana Corpus-Quiguanás, Edna Johana Mondragón-Sánchez

**Affiliations:** 1 Docente de la Facultad de Enfermería,Universidad El Bosque.lbernalo@unbosque.edu.co Universidad El Bosque Facultad de Enfermería Universidad El Bosque Colombia lbernalo@unbosque.edu.co; 2 DocenteUniversidad del Quindío,Armenia,Colombia.jcorpus@uniquindio.edu.co Universidad del Quindío Universidad del Quindío Armenia Colombia jcorpus@uniquindio.edu.co; 3 DocenteUniversidad del Quindío, Armenia,Colombia.ejmondragon@uniquindio.edu.co Universidad del Quindío Universidad del Quindío Armenia Colombia ejmondragon@uniquindio.edu.co

**Keywords:** Primary Health Care, Primary Prevention, Nurses, Professional Role, Professional Practice, Atención Primaria de Salud, Prevención Primaria, Enfermeros y Enfermeras, Rol Profesional, Práctica Profesional, Cuidados de Saúde Primários, Prevengáo Primária, Enfermeiros, Papel Profissional, Prática Profissional

## Abstract

**Introduction:**

Primary health care is a strategy that involves the different health professionals and other agents in the system who can influence the social determinants that affect human wellbeing.

**Objective:**

To know the meanings about primary health care of nursing professionals practicing in this field in a municipality of Colombia.

**Materials and Methods:**

Qualitative, exploratory, descriptive study. The research was conducted in a State Social Company (ESE for its acronym in Spanish) that provides primary health care services. Data was collected through semi-structured interviews. The sample consisted of 13 professionals. IRaMuTeQ software was used to help data interpretation.

**Results:**

‘Nurse’ was the most frequent word. The content analyzed was categorized into four classes:‘Meanings about primary health care’, ‘nursing activities in primary health care’, ‘barriers encountered in primary health care’, ‘primary health care organization and work team’.

**Conclusions:**

Primary health care meaning is understood as the entry point of users into the health system and the one responsible for coordinating their passage through other points of the healthcare network according to users’ health needs. In addition, primary health care is understood as the level of care at which essential health actions focused on the early detection of health risks and complications are carried out, so that health interventions can be planned, implemented, and evaluated on that basis.

## Resumen

**Introducción:**

La Atención Primaria en Salud es una estrategia que involucra a los diferentes profesionales de salud y demás actores del sistema que puedan influir en los determinantes sociales que afectan el bienestar del ser humano.

**Objetivo:**

Conocer los significados sobre Atención Primaria en Salud del profesional de enfermería que ejerce en el área en un municipio de Colombia.

**Materiales y Métodos:**

Estudio cualitativo, exploratorio, descriptivo. La investigación se efectuó en una empresa Social del Estado que brinda servicios de Atención Primaria en Salud; la recolección de datos se llevó a cabo mediante entrevistas semiestructuradas. La muestra estuvo conformada por 13 profesionales. Con la finalidad de auxiliar la interpretación de los datos, se utilizó el software IRaMuTeQ.

**Resultados:**

La palabra más frecuente fue “enfermero”. El contenido analizado fue categorizado en 4 clases: “Significados sobre Atención Primaria en Salud”, “Actividades de Enfermería en Atención Primaria en Salud”, “Barreras encontradas en la Atención Primaria en Salud”, “Organización y Equipo de trabajo en Atención Primaria en Salud”.

**Conclusiones:**

El significado de Atención Primaria en Salud es entendido como la puerta de entrada de los usuarios al sistema de salud, y la encargada de coordinar el paso del mismo por otros puntos de la red de atención de acuerdo a la necesidad de salud del usuario, además es comprendida como el nivel de atención donde se realizan las acciones de salud esenciales enfocadas en detectar tempranamente riesgos y complicaciones de la salud, para con base en ello, planear, ejecutar y evaluar las intervenciones en salud.

## Resumo

**Introdujo:**

A Atengáo Primária a Saúde é uma estratégia que envolve diferentes profissionais de saúde e outros atores do sistema que podem influenciar os determinantes sociais que afetam o bem-estar do ser humano.

**Objetivo:**

conhecer os significados sobre Atengáo Primária a Saúde do profissional de enfermagem que atua na área em um municipio da Colombia.

**Materiais e Métodos:**

Estudo qualitativo, exploratório, descritivo. A pesquisa foi realizada em uma Empresa Social do Estado que presta servidos de Atengáo Primária a Saúde; a coleta de dados foi realizada por meio de entrevistas semiestruturadas. A amostra foi composta por 13 profissionais. Para auxiliar na interpretado dos dados, foi utilizado o software IRaMuTeQ.

**Resultados:**

A palavra mais frequente foi "enfermeira". O conteúdo analisado foi categorizado em 4 classes: “Significados da Atengáo Primária a Saúde", “Atuagóes de Enfermagem na Atengáo Primária a Saúde ",“Barreiras encontradas na Atengáo Primária a Saúde "“Organizado e Equipe de Trabalho na Atengáo Primária a Saúde ".

**Conclusóes:**

O significado da Atengáo Primária a Saúde é entendido como a porta de entrada do usuário no sistema de saúde, responsável por coordenar sua passagem por outros pontos da rede de atengáo de acordo com as necessidades de saúde do usuário. nível de atengáo onde sáo realizadas agóes essenciais de saúde com foco na detecgáo precoce de riscos e complicagóes a saúde, a fim de planejar, executar e avaliar intervengóes de saúde com base nisso.

## Introduction

In 1978, during the international conference on Primary Health Care (PHC), this was defined as essential health care based on practical, scientifically founded and socially acceptable methods and technologies available to all individuals and families in the community^1^. Four decades later, the “Astana Declaration” reaffirmed that PHC is the cornerstone of a sustainable health system, having a more pertinent and effective approach regarding the physical and mental health of individuals who make up society; besides amply contributing to the achievement of the sustainable development objectives related with health^2^.

Primary health care is a strategy that involves different health professionals and other actors of the system who can influence on the social determinants that affect human wellbeing. One of the health professionals who must be widely competent on PHC is the nursing professional; who is involved in the entire planning, execution, and evaluation process of the PHC strategy.

The nursing role has been conducted throughout history in different areas, permitting the discipline to increase its knowledge through the foundation of theories and expansion of work skills, which has permitted the International Council of Nurses (ICN) to determine the professional’s fundamental functions in areas of health promotion, early detection of disease, restoration of health, and relief of suffering^3^.

Some authors, like Zug et al., in 2016, identified globally that the professional exercise of nurses in the PHC context can be categorized into three practices: service practices, community practices, and administration and training practices, also concluding that, although it is true that there is a strengthening and advancement of the various PHC strategies, the process is complex and change in the service offer still has not taken place completely; this, derived from several causes, where one of the most relevant is that the vision by the nurse and the health staff is still focused on a biomedical model; however, it is highlighted that the transformation of the Nursing Care Process is occurring in Brazil^4^.

In Colombia, nursing professionals have been immersed in PHC through what is denominated as first level of care or of low complexity; the establishment of different regulations, like Legislation 1438 of 2011 highlights the orientation of a general health social security system within the framework of a PHC strategy and the comprehensive healthcare policy guides the development of said approach. Nevertheless, authors, like Bruno in 2015, indicate that in the country the segmentation of the system and the funding model show disagreements and barriers to comprehend the PHC model integrally for the entire population^5^.

The role of the nursing professional is of interest for the implementation, management and planning of PHC services^6^. However, Bruno highlights that the role of the nurse is unclear for some actors of the health system, such as administrators and service providers, and also identifies the lack ofarticulation of work teams in relation to PHC principles^5^. The development of this strategy proposes an adaptation of the improvement of service provision, and fundamentally in education activities with emphasis on PHC for the professional nursing professionals^7^.

Given the foregoing, this study established the objective of knowing the meanings about PHC of nursing professionals who practice in the area in a municipality of Colombia.

## Materials and Methods

This is an exploratory, descriptive study with a qualitative approach, conducted in a municipality in the coffee-growing region of Colombia. Data collection was carried out from January to March 2020, through individual semi-structured interviews with 13 nursing professionals working in Primary Health Care. Primary Health Care.

The semi-structured interviews were conducted following a script with guiding questions on the work process of the nursing professional in the PHC context, which was submitted to a pilot test. Subsequently, the informed consent form was read and signed, the interviews were recorded in audio equipment for subsequent transcription, each interview had an average duration of 40 minutes and were conducted in comfortable places for the participants, in order to guarantee greater privacy. After collection, the interviews were coded to protect the identity of the research subjects. protect the identity of the research subjects.

The data collected were analyzed by means of the Content Analysis technique, which, according to Bardin, should be based on methodological rigor as a way of not running away from the object of study^8^.

To support in the data interpretation, the study used the IRaMuTeQ (Interface de R pour les Analyses Multidimensionnelles de Textes et de Questionnaires) software version 0.7 Alpha 2, which enables different types of analyses of text data, organizing the distribution of vocabulary in comprehensive and visually clear manner with graphic representations obtained from the analyses performed. Subsequently, the database was stored in Mendeley Data^9^.

Moreover, the software uses lexical analyses to identify and reformulate units of text, which are transformed into Initial Context Units (ICUs) and Elemental Context Units (ECUs), identified through the amount of words, frequency, mean, and hapax number (frequent words). A vocabulary search was made and words were reduced, from their roots (derivation), the dictionary was created from the reduced forms and identifying the active and complementary forms^10^.

This research followed the following steps:

1. Recording and transcription of the interviews;
2. Preparation of the corpus following the IRaMuTeQ guidelines;
3. Analysis of the Descending Hierarchical Classification (DHC) presented in dendrogram form, indicating the lexical classes into which the corpus was divided, from the frequency (f) and the Chi-squared (X2)^10^^)^ .

The ethical principles were based on the basic principles of bioethics marked in all ethical regulations, like beneficence, anonymity, protection of sensitive data, informed consent. The principal laws considered for this study were: the Singapore Declaration, Legislation 1098 of 2006, and Resolution 008430 of the Republic of Colombia. This study was approved by the Ethics Committee of the Faculty of Health Sciences at Universidad del Quindío with resolution N°35 10-10-2018. The researchers are part of the Research Group on Primary Health Care (GIAPS, for the term in Spanish) and the Nursing Research Group of Risaralda (GIER, for the term in Spanish), both recognized by COLCIENCIAS - Colombia.

## Results

Analysis of the sociodemographic data permits understanding the profile of the participants of the present study. The participants were 13 nurses who practiced in PHC in the municipality selected, all were females, the relation of time working in PHC and graduate training is described in Table 1.

**Table 1:** Relation of time working in PHC and graduate training of the participants

Identification	Age in years	Graduate	Time of experience in PHC
E1	57	Specialist in Management in Health Services and Audit	15 years
E2	22	None	1 year
E3	21	None	4 months
E4	57	None	32 years
E5	50	None	20 years
E6	26	None	3 months
E7	21	None	1 year
E8	51	None	16 years
E9	32	Specialist in Epidemiology	9 years
E10	42	Specialist in Health Administration	3 months
E11	36	None	4 years
E12	40	None	3 years
E13	24	None	2 years

Regarding participant age, 61% were between 20 and 40 years, and 38% were between 41 and 60 years.

With respect to the complementary training of the 13 nurses interviewed, graduate formation is low, 23% of the participants has completed specialization-type graduate studies.

As for time working in PHC, it was found that 23% of the nurses interviewed have less than one year of experience, 38% have experience between one and five years, and the remaining 38% have more than five years of experience in PHC.

The general corpus was constituted by 13 texts, separated into 671 Text Segments (TS), with use of 506 TS (75.41%). Some 24,427 occurrences (words, forms or terms) emerged, with 2,928 different words and 1,520 with a single occurrence. The content analyzed was categorized into four classes:

Class 1 denominated “Meanings about PHC”, with 81 TS (16.01%)
Class 2 denominated “Nursing activities in PHC” with 114 TS (22.53%)
Class 3 denominated “Barriers found in PHC”, with 110 TS (21.74%)
Class 4 denominated “Organization and work staff in PHC”, with 125 TS (24.7%).

The classes were generated from X2; Similar evocations (words, forms, expressions) emerge in them, with the most representative ones being evident for each class. The X2 > 3.80 was taken as base because it corresponds to p < 0.05. The most-significant phrases are discriminated according to the significance analysis and will be presented in the discussion of each category.

**Figure1:**
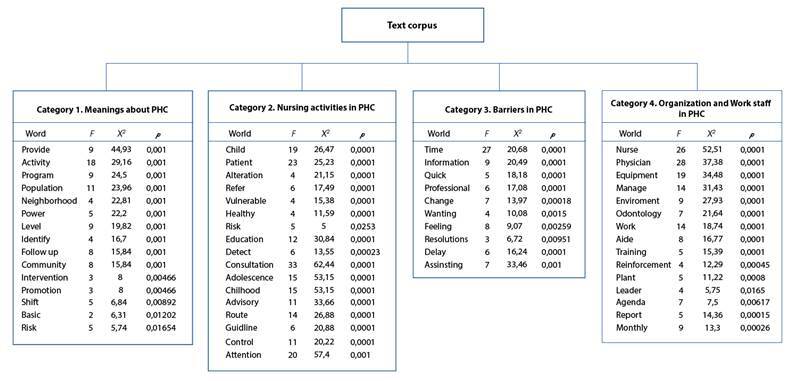
Descending Hierarchical Classification Dendrogram with classes and lists of words per class.

To better visualize the classes, a dendrogram was designed with the list of words from each class generated. Each class indicates the f of each word, the X2 value, and the significance value (p). The following describes, operationalized and exemplified, each of these emergent classes in the Descending Hierarchical Classification(Figure 1).

## Discussion

### Meanings about Primary health care

The discourses of the nurses interviewed show some divergences in the meanings about PHC. about PHC, with a predominance of meanings related to the activities they carry out as professionals professionals:

“*Set of actions that we, from the area, can carry out for the population to identify which are their principal risk factors and modify behaviors that are affecting their health and, besides, learn what is the exact moment to consult the health institutions” E1*

*“.. PHC is the first part when a user arrives, we are like the first face of attention; from there, we then focus on the user if they need a second appraisal by specialists or what we can do to prevent some type of disease..." E2*

*“ ..Primary health care is everything that refers to the care provided to the community of what has to do with the first level of care .not only for them to attend institutions that, well, we see that the volume of these activities is rather low but also looking and going directly to the communities, knowing their needs and bringing them programs to provide care to them.” E12*

Similar findings were reported in a study in the United States on the PHC context, evidencing that some of these meanings were also indicated by the nurses as the motivation when providing PHC and the difference in the lives of their patients^10^.

It should also be highlighted that some narratives by those interviewed show PHC as basic care, referring to the basic term as the essential actions to plan health interventions:

*"Primary, as its name indicates, is the basic, the elementary, it is what is important, it is what constitutes the basis of all health care. as the basis of all health care, and our focus is and should be on promotion and prevention activities..." E4*

*"... APS is the first part when a user arrives, we are like the first face of the attention, and from there we focus on the user if he/she needs a second assessment by specialists or what then we focus on the user if he/she needs a second assessment by specialists or what we can do to prevent any type of disease..."E11*

*"...For me, PHC is, as the word says, the basic care that I can provide to the human being or the person, PHC seeks to reach the community through what, well, through health days, avoiding how to reduce barriers or those care gaps..." E13*

In coherence with the discourses of nurses, PHC is considered as a set of health actions, at the individual and collective level, which includes health promotion and protection, disease prevention, diagnosis, treatment, rehabilitation, harm reduction and health maintenance in order to develop comprehensive care that has a positive impact on the health status of communities^11^^), (^^12^.

It can be inferred that the meanings of some nursing professionals are close to the essential elements that could strengthen the success of the health care system. the essential elements that could strengthen the success of the purposes of the Integrated Health Care Policy^13^.

*"To provide all the actions of promotion and prevention, patient care, community, family, in everything that has to do with promotion and prevention and direct care of the family..." E5*

*"...What PHC is in itself for me is to go to the community, to go and look for an interdisciplinary team that hopefully includes a physician, a nurse, a health promoter, that can be done there in the community where the user is, that can follow up on the risk and everything else that can be done in the community. the community where the user is, to follow up on the risk and all that is required..."E8*

*"... PHC is everything that refers to the attention given to the community in relation to the first level of care... not only for them to attend the institutions, because we see that the volume of these activities is rather low, but also to look and go directly to the communities, to know their needs and to take the programs there in order to provide them with to be able to pay attention to them..." E9*

Nursing, in its current conception, is the reflection of the transformations the health concept has endured over the years; this profession has had consequential actions in the current context with important contributions to the population’s health, a fact that distinguishes it as promotor of human development that produces not only healthcare, but also values and qualities that aim to improve the health of people through PHC.

### Nursing activities in PHC

The participants in the study refer to their work activities as operational management of the health centers; additionally, and during most of their workday, they perform intramural and in some cases extramural nursing consultations (care for individuals at different points in their life course and care for at-risk groups), where they also carry out educational processes.

*"... There are many activities, generally speaking, in early childhood, infancy and adolescence adolescence, I take cytologies, HIV pre-testing, cardiovascular risk, I also coordinate the needs of the health center..."E3*

*“... In the morning it's just me giving education on the use of the glucometer, the patient's pathology, symptoms, warning signs and those given insulin for the first time must also be educated. In the afternoon, the doctor arrives, who also works with us, she is the cardiovascular risk leader; and so, we continue with activities, that the high-cost invoice, the invoices for the EPS and on Tuesdays and Thursdays I go in the morning to the Caimo health Center, and there I do consultation, cytology, first childhood, childhood and adolescence, family planning, pre- and post-HIV.” E6*

Similar results were found in the study by Aguirre in 2018, where nurses with advanced practice in PHC are characterized for spending most of the time in direct contact with patients, followed by the educational processes they carry out, and the lowest fraction of time is dedicated to administration, consulting, and research^14^. Those interviewed never referred to conducting consulting and research processes, which could mark the difference against the advanced nursing practice.

In the participants’ narratives, it was not possible to identify application of the Nursing Care Process (NCP), due to the scope of the interviews, without this meaning non-existence or non-completion of such in their care process. Parra et al., state that one of most important tools for nursing professionals is the NCP because it permits efficient and holistic care of people^15^, given that it is an organized, systematic, dynamic, logical, sequential process divided into five stages (appraisal, diagnosis, planning, execution, and evaluation).

The reports by the nurses interviewed highlight that one of their principal objectives for consultation success is to generate an environment of empathy and trust with users, a fact that allows a glimpse to the use of intrapersonal models within patient care.

*“... here we have access, we are like the first face a user meets, I try to give them a lot of education and give them a lot of trust, so they can tell me how they spend their daily lives, given that often I find reconsulting patients, and we go look, and yes, they are referred to all specialties, but the patient is the same and we see they need a little bit of affection, a little more listening to patients..." E11*

*"...here they already feel more confident with you, depending on the different situations and you see how you can support them". E12*

*"...it seems to me that, if you know how to reach people, the first thing is that people understand and feel you as at the same level and when you know how to reach people, you teach them the physical means for handling a fever, how simple! and speak in the terms that people speak..." E4*

*"... and well when you like it then you know how to reach people and see changes in people thanks to the education that one has given them, that is very satisfying, to see that one's story of a long that is very satisfying, to see that the story you have told them for so long has been accepted..." E8*

Complementing the results found, a study in Spain permitted identifying that health promotion and prevention activities carried out in 14 health centers and by different professionals adhere mostly to intrapersonal models, focused on empowering individuals, guiding the implementation of healthy conducts, early identifying risks and other aspects^16^.

### Barriers found in PHC

From the narratives of the nurses interviewed, barriers emerged of the structural and organizational nature of the services, which hinder work in PHC.

*"... unfortunately, the Ministry is in charge of making changes with the new resolutions, but one sees these changes in which one is more glued to the computer than looking at the patient, it is more and more like that, one spends more time with the clinical history, why? because one has to perform, why? because they are measuring you, because if you do not perform then what happened, why are you not performing?" E7.*

*"... no, it is a process and we are going to see how we are going to continue adjusting, we are going to start reading this new resolution now that it is out again, we are starting to read it, so, everything is a process, we are going to continue adapting, we are going to continue adapting, we are going to see how we are going to adjust. a process, to keep adapting" E10.*

*"... well, for me it is a change, initially I cried, I went and begged the manager to change services, I said no, what a laziness those rules and those resolutions because I had to learn again, besides, other colleagues had told me that I did not make the time, that I was very late in consultations because of the amount of information that had to be asked...". a lot of information that had to be asked ..." E12*

Structural barriers were constituted as changes in the new health care model in the national territory, which have been implemented in the last five years through various resolutions, including: Resolution 429 of 2016, which adopts the Integrated Health Care Policy (PIAS); Resolution 3202 of 2016, which adopts a group of Integrated Health Care Routes; and Resolution 3280 of 2018, which adopts a group of Integrated Health Care Routes. Integral Health Care Routes; and Resolution 3280 of 2018.

Whereby the technical and operational guidelines of the Comprehensive Care Route for the Promotion and Maintenance of Health and the Comprehensive Health Care Route for the Promotion and Maintenance of Health are adopted. for the Promotion and Maintenance of Health and the Integral Health Care Route for the Maternal and Perinatal Perinatal Maternal and Perinatal Population and establishes the guidelines for their operation.

According to the nurses interviewed, with the new health policies, a new challenge was imposed, which refers to unlearning and relearning the new model of care through the updating of knowledge and, consequently, of PHC practices.

Nurses identify current policies as a structural barrier that hinders the adaptation process in the incorporation of these policies in professional practice, as well as limiting the development of a person-centered health care.

Although the aim of the new healthcare model is to guarantee comprehensive care to people, families, and communities from interventions of integral health appraisal^17^, the logic of a reductionist health model still remains, with priority in filling out clinical charts.

According with the nurses, the time assigned for nursing consultations is reduced; given that filling out the clinical chart makes nurses dedicate much of the time of the nursing consultation, with no time left over for other demands or relevant needs detected during the consultation, or to provide adequate education, thus, harming the comprehensive health care proposed by the new model. This leads nursing professionals to a situation of duality in which they must decide which activity to prioritize.

With respect to organizational barriers, one of them, often cited among the nurses interviewed, refers to the time for consultations, which means that nurses must quickly address the needs of users and families, and this, in the perception of those interviewed, compromises the quality of the care provided.

*"... I have an agenda in which, depending on the life course that I manage, for example, if it is early childhood or family planning, there is a time, in that time I have to measure myself. I have a schedule in which, depending on the life course I manage, for example, if it is early childhood or family planning, there is a time, and in that time, I have to measure how long it takes me to see each patient. Sometimes it takes longer than the stipulated time because there are many things to be done to the child...". E2.*

*"... We have already started to implement all the routes, but what we have had the most difficulty with is time, because there are so many things to do and ask questions in such a short time." E4.*

*"... but sometimes, as I said, there is not enough time because there are young people who come and want to know about all the planning methods, so you have to explain everything very well, absolutely everything, and it is very difficult for you to educate them in the consultation time." E6.*

*".It is up to you to take the medical history as quickly as possible because you start to delay the consultation, that part is very negative because then you have to run, fill out everything in a hurry and many times you do not pay enough attention to listen to a patient, but you have to do the survey to fill it out quickly and be able to complete it." E7.*

*".The minimum you would require for a consultation is one hour, they are giving forty minutes, you do not have enough time, what happens to you as a nurse doing a consultation for children, they give you forty minutes and it takes an hour, when you have done three, you are already an hour late, you have to give a moderately good care, when you are an hour late and you still have four more on the agenda, you feel a little crazy, you get stressed, anxious and that is reflected in what? You educated the first ones, but you did not have enough time to educate the last ones." E8.*

*".As such the time of assignment to make these consultations because they are so extensive and they ask so many questions in the clinical history, then the time they gave us before was not enough, they extended it, but it was not enough. They extended it, but even if they extended it, it is not enough time to be able to provide comprehensive care" E9. comprehensive care..." E9.*

In the narratives by the nurses interviewed, the time assigned to carry out nursing consultation is insufficient to conduct care centered on the person, given that the professionals have few or no opportunities to provide integral care that guarantees a holistic approach of the real needs felt by the patient. Qualified listening was perceived by nurse E7 as a necessary practice for a holistic and humanized approach, nevertheless, this practice is harmed by the limited time they have during nursing consultations.

This result was also found by Bruno et al., in a study that sought to determine the PHC knowledge, activities, and barriers in 79 nursing professionals in Barranquilla, Colombia. The authors emphasize that the activities the nurses predominantly carry out are those corresponding to management of clinical charts and appraisal of their information (97%); further, the activities they conduct with lesser frequency are those characteristics of PHC, like promotion and prevention activities for individuals, families, and collectivity (33.3%)^5^.

### Organization and work staff in PHC

Regarding the conformation of the work teams, the participants state having a multidisciplinary team comprised by physicians, odontologists, nurses, odontology aides, nursing aides, security, and billers.

*".Well, my work team is made up of two full time doctors, many times a back-up doctor comes in to cover because obviously the demand for patients is too one of them comes as a backup to cover because obviously the demand of patients is too many for two doctors... I have two billers... I have a filter assistant who is the one who helps me I have two billers ... I have the filter assistant who is the one who collaborates with me in induced demand ... the orderly who is in charge of the entrance; the auxiliary nurse and laboratory." E2*

*“.My work staff includes two staff doctors, one full time which is nine hours and one part time which is four hours; the outpatient technical direction has an assistant, I have two dentists, one for four hours and one for five hours and also a reinforcement twice a week of a dentist for four hours, we have a nine-hour oral hygienist who twice a week goes out extramural to conduct his activities and three intramural; we have a vaccinator in charge of the vaccination program, I have a security guard, an operator, a dentistry assistant, a filter assistant in charge of assigning appointments and being aware of the consultation and substitutions of the doctors and nurse and a biller..” E5*

*“.My work team is very large, in reality there is a lot of staff here, among them we have doctors, specialists, nursing assistants, billers, respiratory therapists, social worker, psychologist, head nurse, so it is a multidisciplinary team, in terms of head nurse I am the only one, there is also a professional nurse who is also the one who is now managing the cardiovascular risk program" E6.*

This result coincides with the findings of a systematic review that included referent countries in PHC, like Brazil, Canada, Chile, Cuba, Ecuador, Spain, and Peru, which highlighted that the conformation of PHC teams is diverse, affirming that these are minimally comprised by a nurse and physician; and according to the health approach in each country, different professionals and technicians in the area are added^18^.

In the discourse of the nurses interviewed, the consolidation of work teams where communication and collaborative work is a fundamental tool to achieve goals and maintain adequate interpersonal and work relationships among PHC team members was evidenced.

*". ..I feel that the relations between us are good, in fact I have all my affections in this work team, I love them very much, I recognize their work because it is really a very judicious work team and among them, there are good relations, that is what I can perceive in what they do, they are very supportive when there is someone who has a lot of work and someone else does not have much, come what helped, when there is not much to do there they come, what is there to do, I am unemployed at the moment. and the attitude towards customer service also seems to me to be very good, I feel very satisfied with my work team." E1*

*"The relationship between them is very communicative, I try to help them ... we have a very good work team, to be honest. We have a very good work team" E2*

*".That all the staff that we are constituted as a work team, that we meet some goals that we have assigned. goals that we have assigned; then for those goals I, for example, as an administrative activity, every two months I have a staff meeting with my work team." E4.*

*".We have been together for a while now, most of us are employees, so it is a very cool environment. I think that one of our greatest strengths is that the work environment is very pleasant, warm and respectful. respectful, but I insist that everyone does their part and does it well, that we all respect each other, that we all help each other. that we all respect each other, that we all help each other, that it is everyone's health center, the work environment is very cool." E4*

Regarding the aforementioned, Alvarez et al., in 2012 refer to the importance of creating PHC teams with conflict resolution skills, effective communication, active participation by all the staff members, highlighting the importance and transcendence of each of them in fulfilling the goals, along with the support that can be provided among the different staff members to reach said goals^19^.

The professionals interviewed permit identifying in their narratives the importance of being coordinators with recognized listening and leadership skills to be able to guide the participants from their work staff in reaching the institutional objectives; for them to generate an environment of trust is an essential element to strengthen the work environment and the performance of officials.

*"..I think I have a good work team, we also realize at the end of the goals, what is happening to us, so that's where we have the meeting and then we look to see because many times it is with discouragement, with the staff, so I try to talk to each one, see if we have this difficulty, how we can improve it, but in itself I think I have a good team and I give them the confidence to come and tell me their concerns."E11*

*"At the moment the work team is excellent, we have good chemistry with me, we work to the extent that each one can We work to the extent that each one can perform his or her functions, each one has specific functions, communication is very good" E5*

An observational study conducted in Colombia states that nursing professionals in PHC have communication and leadership skills, based on their empirical and scientific knowledge, on relations of empathy, values, and individual qualities; skills that allow nurses to be referent and support for their work staff, managing to also influence positively the staff’s dynamics^20^.

## Conclusions

Primary health care is understood as the entry way for users to the health system and that in charge of coordinating their passage through other points of the care network according to users’ health needs. It is also understood as the level of care where essential health actions take place focused on early detection of health risks and complications to - based on such - plan, execute, and evaluate health interventions.

Nursing professionals carry out innumerable activities in their work in PHC, however, the nursing consultation during the different courses of life, like first childhood, childhood, adolescence, youth and adulthood, as well as, educational activities aimed at instructing about healthy conducts, detection of warning signs, management of pathologies, and pharmacological treatment were the activities that stood out in the nurses' narrative, as those performed most frequently in their daily work.

The principal barriers identified during the nursing work process in PHC were, on the one hand, the transition process between the model and former health care policies to consolidate the new Comprehensive Health Care Model and its respective policies, which implies time-consuming deconstruction and relearning processes. On the other hand, the time barrier was identified, given that it is not sufficient to conduct comprehensive care and approach of users and their needs. Lastly, the health staffwho make up the PHC in the municipality studied are multidisciplinaryteams, in whom the need to practice assertive communication and collaborative work are highlighted as fundamental actions for work in PHC.

Although this study had limitations, given that it used only qualitative techniques and the sample size is small, the results may be useful to establish a baseline about the meanings about primary health care in nursing professionals practicing in the area.
